# Psychological distress over 12 months post-diagnosis in an early inflammatory arthritis cohort

**DOI:** 10.1093/rheumatology/keae276

**Published:** 2024-05-15

**Authors:** Lucy Zhao, James Galloway, Jo Ledingham, Sarah Gallagher, Gerasimina Garnavos, Paul Amlani-Hatcher, Nicky Wilson, Lewis Carpenter, Kirsty Bannister, Sam Norton

**Affiliations:** Institute of Psychiatry, Psychology & Neuroscience, King’s College London, London, UK; Center for Rheumatic Diseases, King’s College London, London, UK; Wolfson Centre for Age-Related Diseases, King’s College London, London, UK; Department of Psychology, King’s College London, London, UK; Center for Rheumatic Diseases, King’s College London, London, UK; Rheumatology Department, Portsmouth Hospitals University NHS Trust, Portsmouth, UK; National Early Inflammatory Arthritis Audit (NEIAA), British Society for Rheumatology, London, UK; National Early Inflammatory Arthritis Audit (NEIAA), British Society for Rheumatology, London, UK; National Early Inflammatory Arthritis Audit (NEIAA), British Society for Rheumatology, London, UK; Center for Rheumatic Diseases, King’s College London, London, UK; Department of Psychology, King’s College London, London, UK; Wolfson Centre for Age-Related Diseases, King’s College London, London, UK; Center for Rheumatic Diseases, King’s College London, London, UK; Department of Psychology, King’s College London, London, UK

**Keywords:** early inflammatory arthritis, mental health, patient-reported outcomes, quality of life, longitudinal study

## Abstract

**Objectives:**

People with inflammatory arthritis (IA) experience worsened mental wellbeing alongside disease progression. Using the National Early Inflammatory Arthritis Audit (NEIAA), we assessed trends in psychological distress during the 12 months following IA diagnosis, mapping these against clinical outcomes to identify associations.

**Methods:**

This is a prospective study of people recruited to NEIAA receiving an IA diagnosis and completing the baseline patient survey. Patient-reported outcomes (PROs) at baseline, 3 months and 12 months were collected, including psychological distress [assessed using Patient Health Questionnaire Anxiety and Depression Screener (PHQ4ADS)]. Mixed effects linear regression models estimated associations between predictor variables with psychological distress at baseline and over time.

**Results:**

Of 6873 eligible patients, 3451 (50.2%) showed psychological distress at baseline. Of those completing follow-ups, 30.0% and 24.1% were distressed at 3 months and 12 months, respectively. Higher psychological distress at diagnosis was more commonly reported by younger, female and non-White patients. Clinical factors, including higher counts of comorbidities, prior depression and higher disease activity at diagnosis were associated with higher distress. Higher distress at baseline was associated with poorer outcomes over time in quality of life, disability, work performance, disease activity, as well as reduced likelihood of achieving good treatment response by EULAR criteria.

**Conclusion:**

Half of patients with IA show significant mental health comorbidity at presentation, which associated with worse disease outcomes and quality of life. Screening for anxiety and depression should be a universal standard, and access to effective mood therapies alongside arthritis treatments is essential. Strategies should be culturally valid and consider multi-morbidities.

Rheumatology key messages

- Around half of NEIAA enrollees who completed PROMs reported high psychological distress at diagnosis.
- Psychological distress reduced by 12 months but remained high, affecting around one in four patients.
- Patients with higher psychological distress at diagnosis experienced worse disease and quality of life over time.

## Introduction

Inflammatory arthritis (IA) encompasses a range of conditions. The most prevalent form is rheumatoid arthritis (RA), which affects ∼1% of the adult population [1]. Treatment strategies for IA have historically focused on synovitis and systemic inflammation, with disease-modifying anti-rheumatic drugs (DMARDs) being effective in suppressing local joint inflammation and subsequent pain [2, 3]. However, the psychological impact of the disease remains a frustrating unmet need for patients [4–6].

In IA patient cohorts, the prevalence of depression is at least twice as high as that of the general population. In patients with RA or psoriatic arthritis (PsA), around 20% meet diagnostic criteria for major depressive disorder, and 30–40% experience high levels of distress including depressive and anxiolytic symptoms [7–9]. This high rate of psychological distress can be attributed to a diverse range of factors, including the impact of the diagnosis and concerns about future health [10], disability due to symptoms (e.g. joint stiffness and pain) [11], reduced social roles [12], reduced quality of life (QoL) and work impacts [13]. Furthermore, in addition to indirect impacts, there is increasing evidence that inflammatory processes may be a direct cause of depression [14].

The association between mental health and IA is complex and bidirectional. Psychological distress often impairs patients’ ability to function and cope with their symptoms and is associated with worse disease control and increased disability. This can lead to a vicious cycle of worsening disability, poorer treatment outcomes and mental health, which all reinforce each other to exacerbate impacts on QoL and work impairment [15, 16]. Worse treatment adherence and outcomes have also been shown to correlate with high distress levels [17].

To develop effective strategies to better support affected patients, it is vital that we understand the gravity of the issue. However, few studies have captured the severity of mental health early in the disease course in patients with IA. We aim to show the burden of psychological distress around the time of diagnosis and over 12 months of follow-up in a large contemporary dataset representative of England and Wales. We also aim to describe the mental health impact on patients’ QoL, general disability and work performance.

## Methods

### Study population

The National Early Inflammatory Arthritis Audit (NEIAA) is an observational cohort study of patients (aged ≥16 years) referred to secondary care rheumatology services across England and Wales with suspected early IA (onset <12 months). Patients with a confirmed early IA diagnosis are eligible for follow-up, which takes place at 3 months and 12 months and includes clinical information and a patient-reported outcome (PRO) survey. Our inclusion criteria were NEIAA enrolment between 13 May 2018 and 5 October 2022, and a diagnosis of RA, PsA or undifferentiated arthritis (UA). During this period, 19 325 patients received diagnosis of RA, PsA or UA and were eligible for follow-up. Of these, 6873 were invited and returned the baseline PRO survey and were included in our analysis. Please refer to the 2023 NEIAA annual report on full methodology and cohort design [18].

### Measures

#### Sociodemographic descriptors

Our data analysis included sociodemographic descriptors of the patient cohort, including age, gender and ethnicity, as reported at baseline. Age was included as continuous. Gender included patients reporting as female or male. Ethnicity subgroups were coded as patients reporting as White, Black, Asian, mixed and other ethnicities, but were not distinguished in the analysis due to insufficient sample size per subgroup, and thus ethnicity was analysed as a binary variable indicating White and non-White patients.

#### Clinical descriptors

Clinical variables included in the analysis were working diagnosis, seropositivity, physical health comorbidities and prior diagnosis of depression. Working diagnosis included seropositive RA, seronegative RA, PsA and UA. The weighted total count of comorbidities was scored in accordance with the Rheumatic Disease Comorbidity Index (RDCI) [19], which included all 11 comorbidities, and was coded into groups of none, one or two or more comorbid conditions.

Clinical measures of disease activity were collected at baseline visit, 3-month and 12-month follow-ups. These included DAS28 and its components [swollen and tender joint counts, erythrocyte sedimentation rate (ESR), C-reactive protein (CRP) levels and patient global pain assessment]. We also calculated treatment response at 3 and 12 months based on EULAR response criteria, which classify patients with a DAS28 <3.2 and an improvement of ≥1.2 points as a *good response* [20].

Psychological distress was defined here as a state of emotional suffering characterized by depressive and anxiolytic traits. It was assessed at all three time points using the four-item Patient Health Questionnaire Anxiety and Depression Screener (PHQ4ADS). This combines the two-item versions of the Patient Health Questionnaire (PHQ-2) and the Generalized Anxiety Disorder questionnaire (GAD-2) and has been validated as an ultra-brief screening tool for depression and anxiety with good sensitivity and specificity [21]. PHQ4ADS enquires about the frequency of depressed mood, anhedonia, anxious feelings, and uncontrollable worrying over the past 2 weeks. Scores range from 0 to 12 with higher scores indicating greater symptoms of psychological distress. The cut-off for a positive screen is a score of 3 or higher on either PHQ-2 or GAD-2 [22]. The percentage of people scoring above the threshold was used to indicate the prevalence of psychological distress in the study population.

#### Disability, QoL and work impairment

To examine the impact of IA on work and life, our analysis included PROs of QoL, disability and work performance. QoL was measured by the Musculoskeletal Health Questionnaire (MSKHQ), which captures several aspects of musculoskeletal health (e.g. pain, fatigue, physical function) and the condition’s impact on daily life, where higher scores indicate better QoL [23]. Functional disability was measured by Health Assessment Questionnaire (HAQ), which captures impairment of functional status affected by IA (e.g. mobility, dressing, washing, etc), where higher scores indicate more severe disability [24]. Work impairment was measured by the Work Productivity and Activity Impairment (WPAI) questionnaire, which captures three main aspects of work productivity loss due to health conditions, including absenteeism (work time missed), presenteeism (reduced at-work effectiveness) and overall impairment (global activity impairment) [25]. Higher scores on each component indicate greater impact of the condition.

### Statistical analyses

Patient characteristics were summarized and tabulated based on demographic and clinical descriptors. Continuous data were presented as means and standard deviation (SD) unless data distribution is heavily skewed. Categorical data were presented by absolute numbers and percentages. Significance threshold was *P* <0.05 for cross-sectional comparisons and *P* <0.10 for tests of interaction. The 0.5-SD change was used as a distributional estimate for calculating minimal clinically important differences (MCIDs). All statistical analyses were performed and plotted in Stata/MP 17 (StataCorp LLC, Texas, USA).

Longitudinal linear mixed-effects regression models were estimated to determine the association between baseline clinical and demographic variables and mental health over time. This model allowed for simultaneous examination of cross-sectional (baseline) and longitudinal associations between baseline predictor variables and outcome variables over time, using a random-intercept to account for the repeated assessments within individuals. Psychological distress was included as the outcome variable and predictor variables included age, gender, ethnicity, working diagnosis, comorbidity count, history of depression and baseline DAS28 in their respective models. All models controlled for age, gender, ethnicity and working diagnosis. In addition, data collection during the COVID-19 pandemic (after 1 March 2020) was included as a dummy-coded variable (i.e. before and during pandemic). Assessment time-point was included as a dummy-coded covariate (i.e. 0 month, 3 months, 12 months). Interaction terms between predictor variables and dummy-coded time covariates allowed for estimation of differential effects of the predictors on mental health at each time point. The model accounts for missing data based on the missing-at-random assumption.

Further longitudinal linear mixed-effects models were estimated with psychological distress included as a predictor variable, and outcome variables included DAS28, treatment response, MSKHQ, HAQ and WPAI. All models controlled for age, gender, ethnicity and working diagnosis. Predicted marginal means were plotted for all outcome variables, except for treatment response, which was displayed as predicted relative odds of a good treatment response.

Although DAS28 is typically only used for patients with RA, NEIAA collected DAS28 components from all patients eligible for follow-up. Therefore, we performed sensitivity analyses in the relevant models by comparing models excluding or including patients without RA diagnosis. To further examine the influence of changes in DAS28 on psychological distress, a simple linear regression was performed to calculate the proportion of the variation in PHQ4ADS scores explained by changes in DAS28 (R^2^) at 3-month and 12-month follow-up for complete cases.

### Ethical approval

We received approval from the Health Quality Improvement Partnership (HQIP) to conduct research in NEIAA, which has permission from the UK Government Secretary of State for Health to collect for audit purposes.

## Results

The analysis included a total of 6873 patients with either seropositive RA (*n* = 3446), seronegative RA (*n* = 1406), PsA (*n* = 932) or UD (*n* = 778). The average age was 58 years, and female patients (62.8%) and White patients (91.4%) were the majority. Key demographic and clinical data are outlined in Table 1. As shown in Table 2, the prevalence of patients with positive PHQ4ADS scores decreased from 50.2% at baseline to 30.0% at 3 months, and then to 24.1% at 12 months. Out of a total score of 6, PHQ4ADS scores dropped from an average 4.73 (SD = 3.85) at baseline to 3.12 (SD = 3.42) at 3 months, and then to 2.66 (SD = 3.14) at 12 months. In this cohort, the MCID for PHQ4ADS scores is 1.925 (i.e. a change of 2 points or more is considered clinically meaningful). The average change from baseline to 12 months is 2.1 points; 1229 patients (41.7%) showed clinically meaningful improvement from baseline to 3 months, and 732 (43.0%) from baseline to 12 months.

**Table 1.** Study population demographics

Variable		
Patients, N (%)		6873 (100)
Age, years, mean (SD)		58.0 (15.6)
Gender, N (%)	Male	2554 (37.2)
	Female	4319 (62.8)
Ethnicity, N (%)	White	6282 (91.4)
	Black	92 (1.3)
	Asian	323 (4.7)
	Mixed	29 (0.4)
	Other	117 (1.7)
	Unknown	30 (0.4)
Time of baseline data collection,^a^ N (%)	Before COVID-19 pandemic	4699 (68.4)
	During COVID-19 pandemic	2174 (31.6)
Prior depression, N (%)	Yes	534 (7.8)
	No	6285 (91.4)
Working diagnosis, N (%)	Seropositive RA	3446 (50.1)
	Seronegative RA	1401 (20.4)
	Psoriatic arthritis	932 (13.6)
	Other (undifferentiated arthritis)	778 (11.3)

a Start of COVID-19 pandemic was defined as 1 March 2020.

**Table 2.** Mental health burden at 0 months (baseline), 3 months and 12 months

Visit (month)	Prevalence of screened positive			PHQ4ADS mean (SD)		
	PHQ4ADS (≥3)	PHQ2 (≥3)	GAD2 (≥3)	PHQ4ADS	PHQ2	GAD2
0	50.2% (*N* = 3451)	45.7% (*N* = 3143)	34.5% (*N* = 2371)	4.7 (3.85)	2.6 (2.05)	2.1 (2.07)
3	30.0% (*N* = 883)	26.4% (*N* = 777)	20.1% (*N* = 593)	3.1 (3.42)	1.7 (1.85)	1.4 (1.78)
12	24.1% (*N* = 410)	20.2% (*N* = 344)	17.4% (*N* = 296)	2.6 (3.14)	1.4 (1.69)	1.2 (1.63)

### Psychological distress and baseline sociodemographic and clinical factors

**Figure 1.**
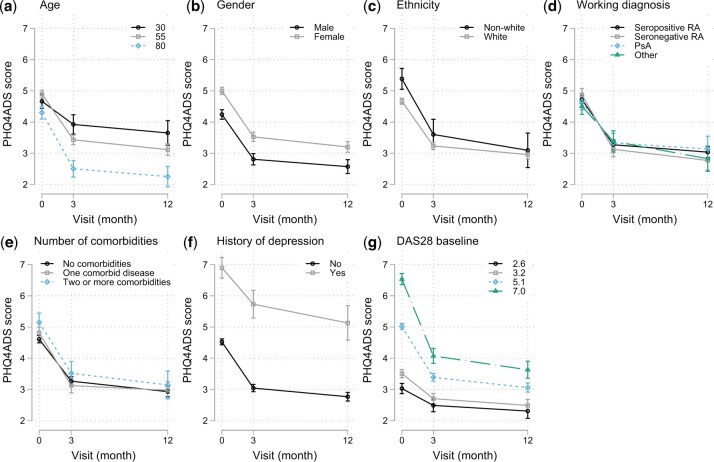
Associations between psychological distress and baseline sociodemographic and clinical descriptors. Higher psychological distress at baseline associated significantly with patients who were younger, female and non-White as well as those who with seronegative RA, had a prior diagnosis of depression and higher baseline DAS28. Over time, a sharper decrease in psychological distress was associated with older ages, non-White ethnicity, higher comorbidity count and higher baseline DAS28. PHQ4ADS: Patient Health Questionnaire Anxiety and Depression Screener (4-items); RA: rheumatoid arthritis; PsA: psoriatic arthritis; DAS28: disease activity score

As shown in Fig. 1 and Supplementary Table S1, available at *Rheumatology* online, our cross-sectional analysis using mixed-effects linear regression revealed that psychological distress associated significantly with younger age (β-coefficient = 0.07, *P *< 0.01, 95%CI = [0.03, 0.10]), the female gender (β-coefficient = 0.72, *P *< 0.001, 95%CI = [0.52, 0.91]), White ethnicity (β-coefficient = −0.61, *P *< 0.01, 95%CI = [−0.97, −0.26]), seronegative RA (β-coefficient = 0.31, *P *< 0.01, 95%CI  = [0.07, 0.55]), higher comorbidity count (β-coefficient = 1.00, *P *< 0.01, 95%CI = [0.66, 1.34]), prior depression (β-coefficient = 2.30, *P *< 0.01, 95%CI = [1.95, 2.65]), and higher baseline DAS28 (β-coefficient = 0.80, *P *< 0.01, 95%CI = [0.73, 0.86]). Notably, age was included as a quadratic variable as it had a non-linear association with psychological distress (Supplementary Fig. S1, available at *Rheumatology* online). Specifically, we found that patients before approximately the age of 50 reported higher psychological distress than those after. No significant correlations were found between PHQ4ADS scores, or percentage screened positive and time of data collection (before/during the COVID-19 pandemic).

Our longitudinal analysis showed that the dynamic trajectory of psychological distress was significantly influenced by age, ethnicity, comorbidity count and baseline DAS28. Specifically, a sharper decrease in psychological distress was associated with older ages (3-month: β-coefficient=−0.06, *P* <0.05, 95%CI=[−0.10, −0.01]; 12-month: β-coefficient=−0.06, *P* <0.05, 95%CI=[−0.13, −0.003]), non-White ethnicity (3-month: β-coefficient = 0.55, *P* <0.05, 95%CI=[0.05, 1.05]; 12-month: β-coefficient = 0.78, *P* <0.05, 95%CI=[0.19, 1.37]), and higher baseline DAS28 (3-month: β-coefficient=−0.44, *P* <0.05, 95%CI=[−0.52, −0.35]; 12-month: β-coefficient=−0.50, *P* <0.05, 95%CI=[−0.60, −0.39]).

### Baseline psychological distress and reported measures of QoL, disability and work

**Figure 2.**
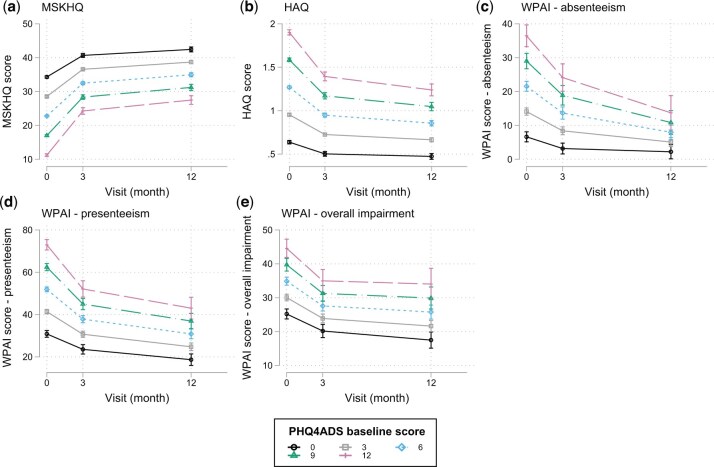
Associations between baseline psychological distress and patient-reported outcomes on QoL, general disability and work quality. Higher psychological distress at disease onset associated significantly with poorer outcomes in MSKHQ, HAQ and WPAI components (absenteeism, presenteeism and overall impairment). PHQADS: Patient Health Questionnaire Anxiety and Depression Screener; MSKHQ: Muskulokeletal Health Questionnaire; HAQ: Health Assessment Questionnaire; WPAI: Work Productivity and Activity Impairment questionnaire

As shown in Fig. 2 and Supplementary Table S2, available at *Rheumatology* online, we found that higher baseline psychological distress correlated significantly with lower scores of MSKHQ (β-coefficient=−1.92, *P* <0.05, 95%CI=[−1.98, −1.87]), lower scores of HAQ (β-coefficient = 0.11, *P* <0.05, 95%CI=[0.10, 0.11]), and lower scores of WPAI components at baseline (absenteeism: β-coefficient = 2.47, *P* <0.05, 95%CI=[2.15, 2.83]; presenteeism: β-coefficient = 3.51, *P* <0.05, 95%CI=[3.22, 3.80]; overall impairment: β-coefficient = 1.62, *P* <0.05, 95%CI=[1.31, 1.92]). A slower improvement of these scores was also associated with higher psychological distress reported at baseline, except for reported overall impairment scores at 12 months, which had overlapping confidence intervals.

### Psychological distress and disease activity

**Figure 3.**
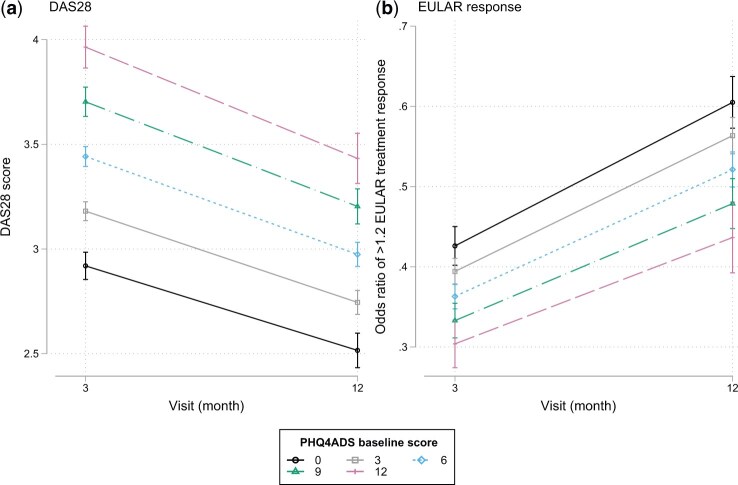
Associations between baseline psychological distress and disease activity and treatment response. Patients reporting worse mental well-being at baseline reported more severe disease activity over time (**a**) and were less likely to present a good treatment response based on EULAR criteria (**b**)

As shown in Fig. 3, our cross-sectional analysis revealed that higher baseline psychological distress was significantly associated with higher DAS28 (3 months: β-coefficient=0.09, *P* <0.001, 95% CI=[0.08, 0.10]; 12-month: β-coefficient = 0.08, *P* <0.001, 95% CI=[0.06, 0.09]) and lower likelihood of a good treatment response (3-month: β-coefficient = 0.94, *P* <0.001, 95% CI=[0.92, 0.96]; 12-month: β-coefficient = 0.92, *P* <0.001, 95% CI=[0.89, 0.95]). In patients reporting higher psychological distress, the odds of a good EULAR treatment response (≥1.2 change in DAS28) were 10% lower than that in patients reporting lower distress (odds ratio: 0.9:1), which remained consistent at 3-month and 12-month follow-ups.

**Figure 4.**
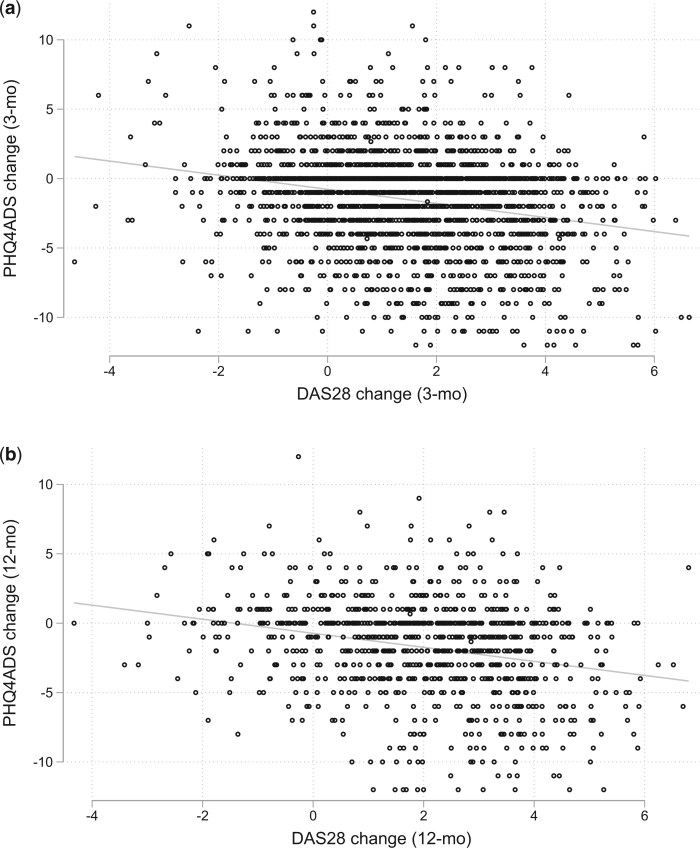
(**a**) Correlation between changes in DAS28 and PHQ4ADS (0–3 months). DAS28 accounted for 5.8% of the variance in PHQ4ADS from 0 to 3 months. (**b**) Correlation between changes in DAS28 and PHQ4ADS (0–12 months). DAS28 accounted for 6.5% of the variance in PHQ4ADS from 0 to 12 months

We examined whether DAS28 was the main contributing factor for the overall reduction in psychological distress levels by performing simple linear regression between DAS28 changes and PHQ4ADS changes over time. As shown in Fig. 4, DAS28 accounted for 5.8% and 6.5% of the variance in PHQ4ADs during the 0–3 month and 0–12 month periods, respectively.

## Discussion

Poor mental well-being is prevalent in patients with IA and remains a clinically unmet problem. The prevalence and magnitude of psychological distress in IA is important as it is associated with lower QoL, worse health outcomes, as well as higher costs and use of healthcare resources [9, 10, 12, 26]. In this cohort with early IA, psychological distress was highest and most prevalent at baseline and decreased over time to moderate levels at 12 months after initial diagnosis.

This is consistent with other early IA cohorts [27]. Diagnosis of chronic illness is potentially distressing and the finding that it is highest around the time of diagnosis, at a time when treatment has yet to be initiated, is unsurprising. However, the magnitude of the prevalence of distress in this cohort is concerning. We observed the improvement was more dramatic in the first 3 months of observation. This likely reflects a process of psychological adjustment for patients, as they have time to process their diagnosis and adapt to changes in their lifestyle [9, 28], as well as initiate DMARDs that effectively suppress inflammatory symptoms and reduce pain and disability [29]. Although large improvements in mental health symptoms are observed in our study, it is important to bear in mind that psychological distress remains high at 12 months, with one in four patients experiencing clinically significant levels; three to four times higher than general population levels. While 12 months is a relatively short window for resolution, we acknowledge that previous research has demonstrated trajectories of distress are generally set within this period, and likely to remain an unmet need over longer periods [29].

### Psychological distress and age, gender, ethnicity and comorbidity

IA is often accompanied by distressing symptoms such as pain and fatigue, which can persist despite intensive DMARD treatments [30]. These underlying symptoms may partly explain the consistent levels of mild to moderate psychological distress at 3-month and 12-month follow-ups. It is becoming increasingly evident that appropriate stratification of the patient population is needed to identify patients at risk for developing mental health issues, as this could help tailor care and optimize treatment. In this study cohort, we identified sociodemographic descriptors, including age, gender and ethnicity, as such risk factors, including patients who were younger (<50 years), female or non-White.

Firstly, younger adults often perceive the diagnosis of a long-term condition as more negative, as they are less likely to expect a diagnosis and less accustomed to changes to their regular lifestyles [31, 32]. As musculoskeletal health (particularly of the hands) is one of the earliest aspects rapidly impacted by IA, the loss of physical mobility and dexterity can be upsetting for younger patients; for example, when their engagement and enjoyment in work and hobbies are impaired. As anhedonia is a core construct of depression/low mood, the psychological impact of the condition is potentially exacerbated in the younger patient cohort.

Secondly, the positive association between female patients and higher distress levels may partly reflect higher pain scores and severe functional disabilities reported more frequently in female patients compared with male patients [33].

Thirdly, the higher rates of mental health issues reported in non-White ethnicities may account for the higher distress levels in the non-White groups. However, we cannot overlook the racial disparities regarding treatment goals and disease education within the healthcare system, as shown in the same cohort [34]. Reduced adequate treatment and care undoubtedly can significantly impact the patients’ abilities to manage their symptoms and subsequently their mental wellbeing. This may be one of the underlying reasons explaining the observed higher psychological distress in non-White patients at baseline and over time; however, further research is needed in this area. We acknowledge that ethnic minority groups may be underrepresented in this study (Supplementary Table S3, available at *Rheumatology* online) and note that the dataset was not weighted to correct for biases in response rates in non-White groups and groups reporting high psychological distress, where the follow-up/completion rate was lower [35].

In addition to sociodemographic descriptors, we found that higher psychological distress was more likely in patients reporting more comorbidities, many of which correlate with poor mental health outcomes [36–38]. Specifically, and similar to other studies [39, 40], we found that patients with two or more comorbid conditions reported the highest psychological distress compared with those with one or no comorbidity. This echoes the concept of a syndemic framework, which suggests mental health issues are not driven by a single disease, but rather by the physical and emotional burden of a conglomerate of coexisting conditions, which also predispose patients for new diagnoses of other long-term conditions.

### Psychological distress and disease activity

Our study reveals high psychological distress in a large contemporary national cohort. The high co-prevalence of poor mental health with IA has been well-recorded and many studies have provided insights on the complex bidirectional relationship between mental health and IA. Acute and chronic manifestations of the inflammatory component directly impact well-being, quality of life and work, while mental health issues impair symptom management, creating a vicious cycle [14]. Consistent with other studies, we found that patients with prior depression are particularly prone to high psychological distress in early IA even after remission, which may partly be attributed to treatment complications and poor symptom management [17, 41, 42]. In our cohort, high distress levels at baseline correlate with increased impediments in daily routine and work-related challenges. A qualitative study shows that patients with PsA were already suffering from functional limitations and high levels of distress at the time of diagnosis [43]. Even without underlying levels of psychological distress, diagnosis alone imposes major disturbances. As reported in Rezai *et al.* 2014, ∼30% of patients with RA leave their jobs and 16% change jobs within the first 2 years of diagnosis [15]. As such, major challenges imposed by IA in routine life inevitably impact mental health. However, we did not test whether reported outcomes on disability and work mediate the relationship between psychological distress and disease activity.

In this NEIAA cohort, higher psychological distress at baseline associated with exacerbated disease activity over time and attenuated improvement, yet, inversely, DAS28 accounted for no >6% of the change in PHQ4ADS scores over time. This suggests the involvement of unmeasured covariates and begs the identification of these underlying constructs. Indeed, compelling evidence supports robust connections between depression and inflammation in IA, as depression drives peripheral and central inflammation and several cytokines implicated in depression also play influential roles in IA, including CRP, TNF-*α* and IL-6 [44, 45]. Interestingly, although few studies examine psychological comorbidities in a symptom-specific manner, it is suggested that anxiety may be mediated by different inflammatory processes compared with depression [46, 47]. However, it remains controversial whether immunotherapies can alleviate mental health symptoms or whether antidepressants can suppress pain and inflammation, which implies there remains much to be understood about these complex neuroimmune interactions [48, 49].

Psychological distress often exacerbates pain and fatigue, which are frequently reported by patients as the most distressing IA symptoms. Historically, inflammation has been considered the main source of pain in IA; however, many patients still suffer from moderate levels of pain despite well-controlled inflammation, which we now recognize as centrally mediated non-inflammatory pain [50]. It is believed that non-inflammatory pain can be driven by underlying mental health issues (e.g. depression and anxiety) that induce imbalances in central pain processing that may tip pain modulation in favor of pain facilitation [51]. Psychological interventions, such as cognitive behavior therapy, can alleviate depressive symptoms, pain and disability in RA patients [52]. Therefore, integrating both physical and psychological therapies is crucial in IA treatment, aligning with the National Institute for Health and Care Excellence (NICE) guidelines advocating for mental health management to enhance treatment outcomes [44].

Patients with higher psychological distress may experience worse prognosis and treatment outcomes and are less likely to achieve remission [53]. This may be due to, not only inadequate coping strategies and poor medication adherence, but also social determinants beyond individual control. Lower socioeconomic status can hinder access to health services, as patients with RA are often not exempt from NHS prescription charges. Dey *et al.* 2022 explore social deprivation and disease activity using a syndemic framework to explain the collective impact of a wide range of interacting factors in RA, including mental health, comorbidities and lifestyle choices [54]. One of the contributors to disease severity is the concerning lack of patient advocacy and engagement with healthcare professionals. There are several accounts of patients expressing frustration with the inconsistent and inadequate support for the psychological impact of their condition [55–57]. The resulting lack of health literacy erodes trust between patient and the healthcare system, depriving patients of essential tools to manage their physical and mental health [58].

### Limitations

Firstly, although PHQ4ADS is a well-validated measure of psychological distress symptomology, it is a self-report measure rather than a full diagnostic interview, therefore our prevalence estimates relate to high distress levels rather than diagnosable psychiatric disorders. Secondly, while DAS28 is well-validated in RA, it is used in NEIAA for those eligible for follow-up, which, in addition to RA, include patients with PsA. While DAS28 may be suboptimal as PsA affects a different range of joints [59], we performed sensitivity analyses that demonstrated our findings unlikely to be influenced by this issue. Finally, there may still be a lack of granularity in time as it is unclear whether psychological distress and IA symptoms fluctuate at similar rates. To effectively capture more latent aspects of the disease, more comprehensive measures of mental health and IA as well as high frequency data collection should be implemented in clinic and in research.

## Conclusion

Our results stress the significant mental health burden in early IA and highlight the importance of addressing mental health issues early as they are associated with poorer disease outcomes and quality of life and work. Despite patients struggling with the physical and emotional challenges, most clinics lack active services for mental health support. This issue urgently needs to be addressed. Clinicians should routinely screen for psychological distress and signpost for appropriate treatment. Rheumatologists need regular training in handling common comorbidities. Understanding the limitations of addressing only the inflammatory aspect, comprehensive training for all doctors should encompass an understanding of mental health issues, awareness of available therapies and local services.

## Supplementary material

Supplementary material is available at *Rheumatology* online.

## Supplementary Material

keae276_Supplementary_Data

## Data Availability

Data used in this study were collected for NEIAA and are available on reasonable request to the data controllers at HQIP. All data relevant to the study are included in the article and its supplementary material. All figures and tables in this article are original.
