# Supplementary material for: Psychological distress over 12 months post-diagnosis in an early inflammatory arthritis cohort
Source: Rheumatology (Oxford). 2024 May 15;64(5):2469–78. doi: 10.1093/rheumatology/keae276 (PMC12048071; doi:10.1093/rheumatology/keae276)
Supplement: keae276_Supplementary_Data [file keae276_supplementary_data.zip › keae276_Supplementary_Data/rhe-24-0414-File009.docx]

**Table S1:** Psychological distress and baseline sociodemographic and clinical factors (β-coefficient, p-value, 95% confidence intervals).

| **Predictor variable** | **Baseline visit** | **3-mo visit** | **12-mo visit** |
| --- | --- | --- | --- |
| **Age** | 0.07, <0.0001, [0.03, 0.10] | 0.01, 0.69, [-0.04, 0.06] | 0.001, 0.975, [-0.06, 0.06] |
| **Gender** Male  Female | 0.72, 0, [0.52, 0.91] | 0.59, 0, [0.35, 0.83] | 0.50, 0.001, [0.22, 0.79] |
| **Ethnicity** Non-white  White | -0.61, 0.001, [-0.97, -0.26] | -0.06, 0.81, [-0.57, 0.44] | 0.17, 0.57, [-0.41, 0.74] |
| **Working** Seropositive RA |  |  |  |
| **diagnosis** |  |  |  |
| Seronegative RA | 0.31, 0.013, [0.07, 0.55] | 0.046, 0.759, [-0.25, 0.34] | -0.10, 0.6, [-0.46, 0.27] |
| PsA | -0.033, 0.82, [-0.32, 0.25] | -0.10, 0.582, [-0.45, 0.25] | -0.06, 0.78, [-0.51, 0.38] |
| Other | -0.133, 0.38, [-0.43, 0.16] | 0.09, 0.625, [-0.28, 0.47] | -0.24, 0.293, [-0.68, 0.21] |
| **Comorbidity** 0 |  |  |  |
| **count** |  |  |  |
| 1 | 0.45, 0, [0.21, 0.68] | 0.31, 0.03, [0.0., 0.60] | 0.50, 0.003, [0.17, 0.84] |
| 2 or more | 1.00, 0, [0.66, 1.34] | 0.97, 0, [0.55, 1.40] | 0.93, 0, [0.43, 1.43] |
| **Prior de-** No  **pression**  Yes | 2.30, 0, [1.95, 2.65] | 2.52, 0, [2.06, 2.98] | 2.19, 0, [1.62, 2.77] |
| **Baseline DAS28** | 0.80, 0, [0.73, 0.86] | 0.36, 0, [0.27, 0.45] | 0.30, 0, [0.20, 0.40] |

**Table S2:** Psychological distress and patient-reported outcomes on QoL, general disability, and work quality (β-coefficient, p-value, 95% confidence intervals).

| **Predictor variable** | **Baseline visit** | **3-mo visit** | **12-mo visit** |
| --- | --- | --- | --- |
| **MSKHQ** | -1.92, p<0.05, [-1.98, -1.87] | -1.37, p<0.05, [-1.48, -1.26] | -1.25, p<0.05, [-1.39, -1.10] |
| **HAQ** | 0.11, p<0.05, [0.10, 0.11] | 0.07, p<0.05, [0.07, 0.08] | 0.06, p<0.05, [0.06, 0.07] |
| **Absenteeism** | 2.49, p<0.05, [2.15, 2.83] | 1.75, p<0.05, [1.33, 2.16] | 0.96, p<0.05, [0.42, 1.50] |
| **Presenteeism** | 3.51, p<0.05, [3.22, 3.80] | 2.38, p<0.05, [1.94, 2.82] | 2.03, p<0.05, [1.45, 2.61] |
| **Overall impairment** | 1.62, p<0.05, [1.31, 1.92] | 1.23, p<0.05, [0.85, 1.62] | 1.38, p<0.05, [0.87, 1.89] |

| **Variable** |  | **Patients with baseline PRO**  **(N = 6,873)** | **Patients without baseline PRO**  **(N = 12,452)** |
| --- | --- | --- | --- |
| **Age, mean(SD)** |  | 58.0 (15.6) | 56.8 (16.4) |
| **Gender, N(%)** | Male | 2,554 (37.2) | 4,762 (38.2) |
|  | Female | 4,319 (62.8) | 7,677 (61.8) |
| **Ethnicity, N(%)** | White | 6,282 (91.4) | 10,231 (82.2) |
|  | Black | 92 (1.3) | 374 (3.0) |
|  | Asian | 323 (4.7) | 1,111 (8.9) |
|  | Mixed | 29 (0.4) | 88 (0.7) |
|  | Other | 117 (1.7) | 418 (3.4) |
|  | Unknown | 30 (0.4) | 232 (1.9) |
| **Baseline DAS28, mean(SD)** |  | 4.7 (1.5) | 4.7 (1.5) |

**Table S3:** Demographics and disease activity of patients with and without complete PRO survey at baseline
