# Supplementary figures and images for: Psychological distress over 12 months post-diagnosis in an early inflammatory arthritis cohort
Source: Rheumatology (Oxford). 2024 May 15;64(5):2469–78. doi: 10.1093/rheumatology/keae276 (PMC12048071; doi:10.1093/rheumatology/keae276)

PHQ4ADS score

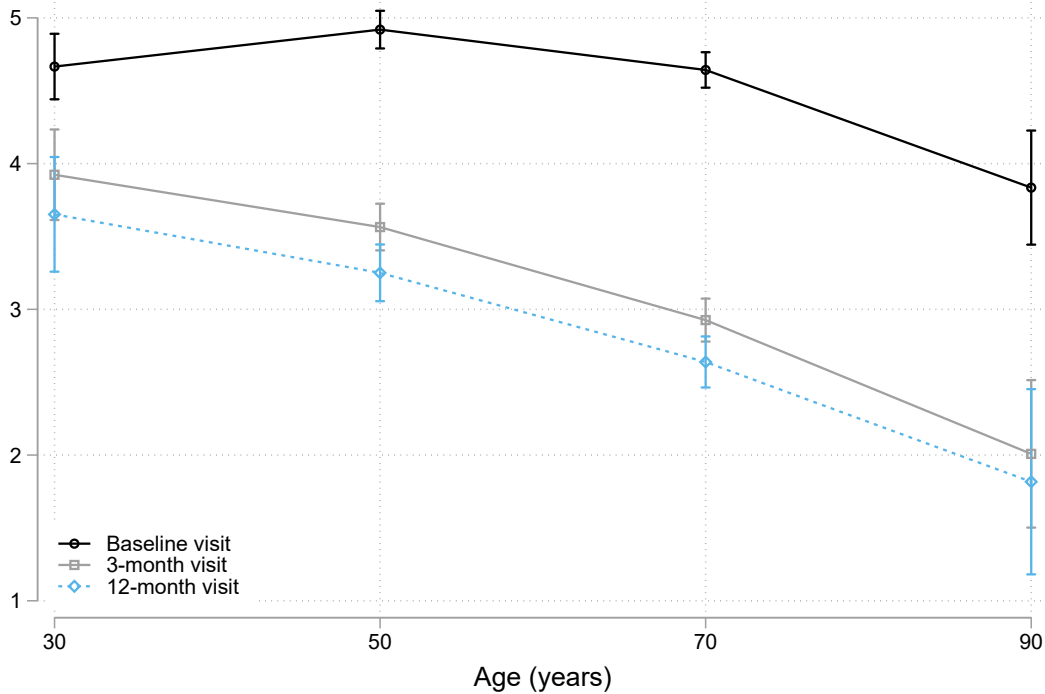

Supplement: keae276_Supplementary_Data [file keae276_supplementary_data.zip › keae276_Supplementary_Data/rhe-24-0414-File007.pdf]
